# A Vein Attempt? Experimental Models for Arteriovenous Fistula Research

**DOI:** 10.1007/s13239-026-00838-w

**Published:** 2026-05-07

**Authors:** Nasir A. Shah, Calvin D. Li, Shannon D. Thomas, Ramon L. Varcoe, Jelena Rnjak-Kovacina, Carmine Gentile, Zoltan H. Endre, Tracie J. Barber, Jonathan H. Erlich, Blake J. Cochran

**Affiliations:** 1 School of Clinical Medicine, Faculty of Medicine & Health, UNSW Sydney, Sydney, NSW Australia; 2 Department of Nephrology, Prince of Wales Hospital, Sydney, NSW Australia; 3 Department of Vascular Surgery, Prince of Wales Hospital, Sydney, NSW Australia; 4 Graduate School of Biomedical Engineering, Faculty of Engineering, UNSW Sydney, Sydney, NSW Australia; 5 University of Technology Sydney, Ultimo, NSW 2007 Australia; 6 Cardiovascular Regeneration Group, Heart Research Institute, Newtown, NSW 2042 Australia; 7 School of Mechanical and Manufacturing Engineering, Faculty of Engineering, UNSW Sydney, Sydney, NSW Australia; 8 School of Biomedical Sciences, Faculty of Medicine & Health, UNSW Sydney, Sydney, NSW Australia

**Keywords:** Arteriovenous fistula, Vascular remodeling, Experimental models

## Abstract

**Background:**

Chronic kidney disease (CKD) affects more than 10% of adults worldwide and is associated with rising mortality and increasing demand for hemodialysis. Hemodialysis requires durable vascular access, with surgically created arteriovenous fistulas (AVFs) being the preferred modality. However, up to 60% of AVFs fail to mature in time for clinical use, whereas others develop excessively high flow that can drive adverse cardiac remodeling and heart failure. These problems reflect incomplete understanding of the biological and biomechanical processes that govern AVF maturation and failure.

**Aim:**

To summarize the experimental models currently used to study AVF maturation and failure—including animal models, computational approaches and in vitro flow systems—and to compare their respective strengths, limitations and complementary roles in vascular access research.

**Summary:**

AVF maturation depends on coordinated arterial and venous remodeling in response to abrupt hemodynamic changes after anastomosis. Altered wall shear stress, pressure and cyclic strain activate endothelial and vascular smooth muscle signaling pathways that promote vasodilation, outward remodeling, matrix turnover and wall thickening. When these adaptive responses are blunted or dysregulated, neointimal hyperplasia, stenosis and access failure ensue. These biomechanical stimuli act within a pro-inflammatory, pro-oxidant uremic milieu, in which circulating toxins impair endothelial function, enhance oxidative stress and bias remodeling towards maladaptation. A broad spectrum of experimental platforms has been developed to interrogate these processes: animal models that recapitulate whole-organism physiology, computational models, including computational fluid dynamics and emerging fluid–structure interaction simulations, that resolve local hemodynamics and wall mechanics; conventional in vitro systems for controlled mechanobiology studies; and emerging microfluidic and macrofluidic devices that impose defined shear waveforms in physiologically relevant geometries. Each model captures selected spatial, temporal or biochemical dimensions of AVF biology, but each is constrained by trade-offs in scalability, fidelity, throughput or translational relevance.

**Conclusion:**

AVF maturation and failure arise from tightly coupled biomechanical and biological interactions, shaped by hemodynamic forces and uremia-related vascular dysfunction. No single experimental platform can encompass this complexity. Progress will depend on systematic comparison of available models and their deliberate integration into a coherent multimodal framework, in which insights from animal studies, computational simulations and in vitro flow systems are used in a complementary manner. Such an approach is essential to identify predictive biomarkers, clarify mechanisms of maladaptive remodeling and guide the rational design of targeted interventions to improve AVF patency and clinical outcomes.

## Introduction

Multiple national screening programs indicate that more than 10% of the adult population exhibit biochemical evidence of chronic kidney disease, CKD [1–3], with the prevalence increasing by 30% over the last 3 decades [4]. In 2019, kidney disease became the 10th leading cause of death worldwide, accounting for ~ 1.3 million fatalities [5]. The number of people receiving renal replacement therapy for end stage kidney disease (ESKD) exceeds 2.5 million and is projected to double by 2030 [6]. Hemodialysis, the predominant treatment modality, requires durable vascular access, with an arteriovenous fistula (AVF) preferred. Successful AVF maturation depends on post-operative vascular remodeling to allow reliable cannulation and adequate blood flow for dialysis. Maturation is considered unassisted if it occurs without endovascular or surgical intervention, and assisted when such interventions are needed [7]. Despite being the gold-standard form of dialysis vascular access, up to 60% of AVFs are not ready for timely use [8]. Patients with AVFs that fail to mature often require prosthetic vascular access, which is associated with higher morbidity and mortality [8]. At the other end of the spectrum, some AVFs remodel excessively and develop high blood flows (>2 L/min) [9]. High-flow AVFs can induce structural and functional cardiac changes, including increased cardiac output, left-ventricular dilation/hypertrophy, right-ventricular remodeling, and elevated pulmonary arterial pressures, increasing the risk of heart failure [10].

After creation, the AVF geometry generates a complex combination of radial, tangential and frictional forces which drive vessel remodeling. The frictional forces generated as blood flows past the endothelium are termed wall shear stress, WSS [11]. Endothelial cells, ECs translate these forces into molecular signals. Laminar flow promotes EC survival and reduces proliferative rate [12], whereas disturbed blood flow, characterized by regions of flow reversal and oscillating WSS, promotes inflammatory gene expression and development of atherosclerosis [13, 14]. These mechanical cues are central to AVF maturation, but they also contribute to maladaptive remodeling when magnitude, directionality, or temporal pattern are unfavorable. Crucially, they act within, and are modified by, the pro-inflammatory uremic milieu of CKD. In this setting, chemotransduction by uremic toxins such as indoxyl sulfate, *p*-cresyl sulfate, and advanced glycation end products alter EC function [15]. Whilst this increases systemic inflammation, oxidative stress, apoptosis, leukocyte migration, thrombosis, and decreases cellular proliferation [16], the net effect of combined mechanical forces and chemical stimuli on AVF remodeling remains poorly defined.

The high rate of AVF dysfunction and associated morbidity reflect an incomplete understanding of the mechanisms governing both adaptive maturation and maladaptive failure after AVF creation. This lack of knowledge has hampered the development of targeted interventions capable of promoting AVF maturation or mitigating maladaptation. This review critically examines current experimental models of AVF biology, with particular emphasis on how they illuminate the mechanisms of maturation, failure, and excessive remodeling, and highlights emerging innovations with the potential to transform the field and improve patient outcomes.

## Physiological Basis of Arteriovenous Fistula Maturation

AVF maturation is governed by dynamic, tightly coordinated biological processes that allow vascular segments to adapt to the abrupt hemodynamic shifts that occur once the vein is surgically anastomosed to the artery. This connection exposes the venous segment to arterial pressure and flow resulting in significant elevations in WSS and transmural pressure which in turn initiate mechanosensitive signaling cascades within the endothelium and underlying vascular smooth muscle cells, VSMC [17].

## Arterial Remodeling

**Fig. 1:**
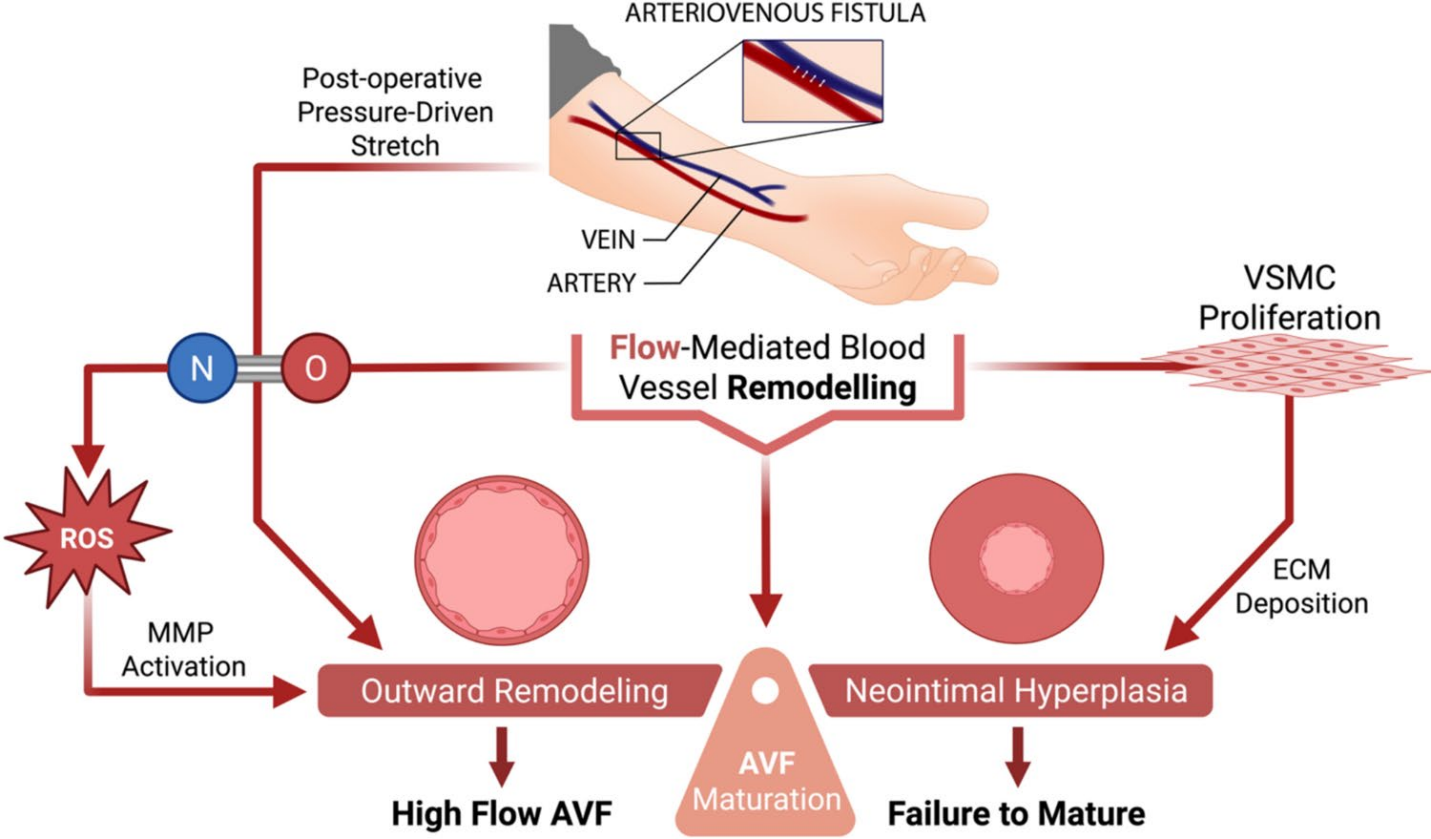
Arteriovenous fistula maturation. Remodeling of the draining vein involves a balance of neointimal hyperplasia (wall thickening), and outward remodeling (vessel dilation). *VSMC* vascular smooth muscle cell, *NO* nitric oxide, *ROS* reactive oxygen species, *AVF* arteriovenous fistula, *ECM* extracellular matrix, *MMP* matrix metalloproteinase. Created in BioRender. Endre, Z. (2025) https://BioRender.com/ddsfwq3

Immediately after creation, the sudden increase in blood flow stimulates ECs to release vasoactive mediators such as nitric oxide (NO), resulting in vasodilation via smooth muscle relaxation [18, 19]. The role of the endothelium in this process is highlighted by the loss of flow-mediated dilation when the endothelium is removed [20]. Arterial dilation, averaging a ~ 20% increase in diameter, buffers elevated WSS, initiating hemodynamic normalization and structural adaptation [21, 22]. A ~ 20% increase in diameter can roughly double theoretical flow capacity. Sustained high flow maintains NO production and activates oxidative signaling [23, 24]. These signals activate matrix metalloproteinases (MMPs), which degrade the extracellular matrix and permit fragmentation of the internal elastic lamina—key steps in enabling continued outward expansion of the vessel [19, 23]. Pharmacological inhibition of either NO synthase or MMPs attenuate arterial remodeling, and endothelial NO synthase-deficient mice show diminished MMP activation and impaired arterial enlargement [19, 23]. Over subsequent weeks, the artery undergoes wall hypertrophy driven by VSMC proliferation and ECM deposition, enhancing tensile strength and maintaining the expanded lumen [22, 25]. Successful AVF remodeling requires a balance of outward expansion and neointimal hyperplasia (Fig. 1).

## Venous Remodeling

Remodeling of the venous segment follows a parallel yet distinct trajectory. Initial venous dilation is largely passive, reflecting the acute rise in luminal pressure. However, sustained high-flow shear stress initiates active, shear-mediated venous remodeling, which can more than double venous diameter [17, 26]. While molecular mechanisms guiding venous remodeling segment are less clearly defined, endothelial NO signaling is likely instrumental [26]. Early eccentric hypertrophy, marked by increased vessel circumference with minimal initial wall thickening, progresses to structural reinforcement via VSMC proliferation and ECM remodeling [27].

Data from the Hemodialysis Fistula Maturation study demonstrated that early postoperative WSS, calculated using CFD, predicted subsequent lumen expansion [28] which is essential to achieve the AVF blood flow required to support hemodialysis. Higher mean WSS on postoperative day 1 was associated with an 11% larger cross-sectional area at 6 weeks and higher WSS at week 6 predicted a larger lumen at 6 months. Conversely, higher oscillatory shear index (OSI), a marker of disturbed flow, correlated with reduced lumen expansion [28]. These observations show that not only the magnitude but also the directionality and stability of flow critically influences remodeling outcomes.

Successful AVF maturation is driven by mechanotransductive pathways including PI3K/AKT/mTOR and Eph-B4/Caveolin-1 (Cav-1) signaling [29]. Cav-1 is crucial for endothelial flow responsiveness and preservation of venous identity with deficiency shown to disrupt Eph-B4 signaling and promotion of pathologic neointimal hyperplasia [30]. In parallel, profibrotic TGF-β/SMAD3 signaling can induce endothelial-to-mesenchymal transition, contributing to neointimal hyperplasia [31, 32]. Inhibition of this axis reduced stenosis and improved vessel patency in large animal models [32]. Inflammatory mediators, such as TNF-α and IL-6, and oxidative stress further compromise remodeling [33, 34]. Increased ROS, particularly superoxide anions, coupled with reduced antioxidant enzyme activity foster endothelial dysfunction and intimal thickening [24]. Although MMP-9 is necessary for adaptive remodeling, upregulation facilitates pathological VSMC migration and maladaptive ECM degradation [35].

Even with optimal surgery, up to 60% of AVFs fail to mature, often due to inadequate outward remodeling or excessive neointimal hyperplasia [29]. Patient factors such as age, sex, diabetes, dyslipidemia, cardiovascular disease, BMI, and dialysis vintage have generally been shown to have no significant association with delayed maturation or primary failure [36]. Across contemporary cohorts, preoperative vessel diameters remain the strongest predictor of successful maturation [36]. Inflammation likely contributes to failure of AVF maturation, but specific comorbidity–outcome linkages remain incompletely defined [37, 38]. The clinical picture is further complicated by excessive remodeling in up to 31% of patients, yielding high flow AVFs [9]. In a multicenter cohort study, a machine learning model using age, first measured AVF flow, diabetes, and dyslipidemia accurately identified patients at risk of developing high flow status, underscoring measurable clinical correlates of an excessive remodeling phenotype [9]. Taken together, these findings suggest that while select clinical variables and early flow characteristics contribute to AVF maturation, they also highlight the absence of a clear mechanistic bridge between known medical co-morbidities and AVF outcomes. A deeper understanding of WSS dynamics, EC and VSMC biology, and the underlying molecular mechanisms is essential for identifying actionable therapeutic targets to improve AVF patency and clinical outcomes in patients on hemodialysis.

## Current Experimental Models

**Fig. 2:**
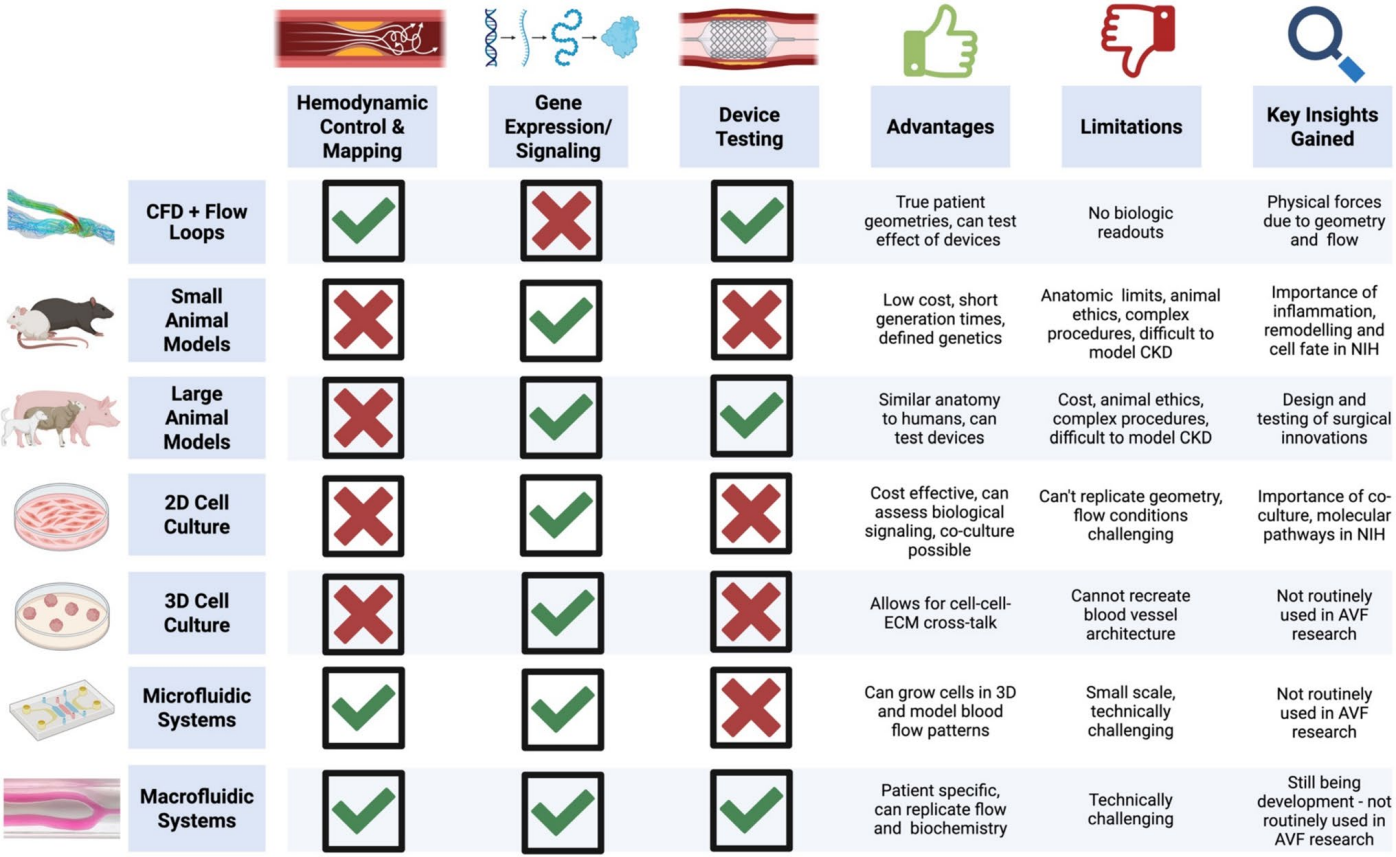
Experimental models in AVF research. *CFD* computational fluid dynamics, *2D* 2-dimensional, *3D* 3-dimensional, *NIH* neointimal hyperplasia, *AVF* arteriovenous fistula, *CKD* chronic kidney disease. Created in BioRender. Endre, Z. (2025) https://BioRender.com/ddsfwq3

Given the practical limits of experimentation in patients, AVF research relies on a set of complementary models rather than a definitive platform. To improve clarity, the following sections are organized according to the principal dimension of AVF biology captured by each approach: whole-organism remodeling (animal models), luminal hemodynamics and wall mechanics (computational and benchtop flow models), and cell-specific mechanobiology (in vitro and flow-based culture systems). For each model class, emphasis is placed on four key questions: what biological or biomechanical process the model interrogates, what major insights it has yielded, what its principal strengths are, and what limitations constrain its translational relevance. Framed in this way, the value of each model lies less in how completely it reproduces the human AVF than in which component of AVF maturation or failure it resolves most effectively (Fig. 2).

### Animal Models

Animal models have been instrumental in elucidating the pathophysiology of AVF maturation and failure. Choice of model is guided by several key principles: (1) preference for non-primate species; (2) humane conduct of all interventions; (3) translational relevance; (4) rapid development of pathology; (5) technical feasibility and reproducibility; (6) capacity for genetic and molecular manipulation; and (7) cost-effectiveness.

### Small Animal Models

Rodents, specifically mice and rats, are the predominant small animal models employed in AVF research [39]. Despite limitations in vascular diameter that constrain detailed hemodynamic analyses, rodent models are especially valuable for mechanistic studies because of their complete genome sequences, extensive homology with humans, and suitability for genetic manipulation [40–42].

Small animal models have been particularly informative in defining the molecular pathways that govern AVF remodeling. In a rat carotid-jugular AVF model arterial wall thickening occurred as early as 1 week post-AVF creation and increased 10-fold by week 3 accompanied by neointimal hyperplasia, luminal narrowing, and venous thrombosis [43]. Histological analyses revealed a predominance of α-smooth muscle actin-positive cells and macrophages expressing membrane attack complex-1 [43]. ROS, particularly those derived from shear stress-induced NADPH oxidase activity (p47phox), were identified as central mediators of flow-induced vascular remodeling with peroxynitrite formation triggering MMP activation and subsequent vessel enlargement and neointimal hyperplasia [23]. Oxidative stress pathways were further explored by Kang et al. who found that heme oxygenase-1 expression conferred vascular protection and promoted adaptive remodeling in a CKD murine AVF model [44, 45], whereas heme oxygenase-1 deficiency increased MMPs expression and impaired AVF function [46]. MMP-9 and β-catenin signaling also contribute to AVF dysfunction with β-catenin activated downstream of endothelial junctional degradation [47, 48]. Notch signaling, a regulator of cell fate and tissue architecture, has also gained interest in AVF remodeling with early inhibition impairing AVF maturation and late inhibition having minimal effect [49].

Rodent models have also clarified the contribution of inflammation to AVF failure. CX3CR1-positive macrophages accumulate in both human and murine stenotic AVFs, with CX3CR1 inhibition reducing neointimal hyperplasia formation and improving AVF patency via suppression of TNF-α and NF-κB signaling [50]. Other studies identified VEGF-A and MMP-9 mediated adventitial fibroblast-to-myofibroblast transition in neointimal hyperplasia, which was attenuated by simvastatin or VEGF-A knockdown [51, 52]. Emerging therapeutic strategies targeting neointimal hyperplasia tested in rodent AVF models include rapamycin-releasing nanofiber scaffolds [53], NO-delivering hydrogels [54], VEGF-A-targeted gene therapy [55], and adipose-derived mesenchymal stem cell therapies [56].

A further strength of rodent models is the ability to simulate CKD. Models have been adapted to simulate CKD using subtotal nephrectomy or adenine-enriched diets. These models have demonstrated that CKD exacerbates AVF dysfunction, leading to delayed remodeling, endothelial injury, increased medial calcification, and enhanced neointimal hyperplasia [24, 44, 45, 47, 49, 51, 52, 57–65]. Langer et al. demonstrated that CKD leads to impaired venous and arterial remodeling, delayed dilation, increased calcification, and heightened neointimal hyperplasia, resulting in AVF stenosis [59]. Liang et al. reported similar findings, with CKD promoting endothelial loss, macrophage infiltration, delayed re-endothelialization, and increased vascular permeability [62]. Taken together, small animal models are most useful for defining causal molecular mechanisms and testing early therapeutic strategies but are less effective for replicating human-scale AVF biomechanics.

### Large Animal Models

Large animal models offer improved fidelity to human vascular physiology and pathobiology [66, 67]. AVFs in these models can be created using human surgical techniques and instrumentation, facilitating real-time assessment of WSS, vessel compliance, and access patency under clinically relevant conditions [68].

Porcine models accurately replicate human vascular dimensions and flow parameters [66] (Table 1). Krishnamoorthy et al. confirmed that regions of low or oscillatory WSS in pig AVFs predicted the spatial distribution of neointimal hyperplasia, echoing patterns seen in human AVF pathology and validating earlier rodent findings in a translationally relevant system [66]. Models in pigs have also demonstrated rapid, consistent intimal hyperplasia and thrombus formation, permitting robust evaluation of interventions to reduce stenosis [68–70]. The longer lifespan of large animals allows longitudinal monitoring of AVF patency, blood flow, and histological changes, allowing modeling of chronic processes such as venous aneurysm formation and late stenosis [71]. Pig models have also been utilized for testing device-based and pharmaceutical interventions, including nanoparticle-mediated delivery of vitamin D, which significantly reduced inflammation and stenosis [65].

Sheep models offer similar advantages, particularly in mimicking the venous architecture of human upper limbs (Table 1). They have been used to test surgical devices and to evaluate maturation dynamics, patency, and histopathology [67, 72]. Sheep AVFs have been instrumental in evaluating surgical innovations such as novel sutureless anastomotic devices, as well as device- and drug-based therapies in a setting that closely mimics human vascular access [67]. Dog models enable long-term studies of stent endothelialization and vascular response to prosthetic implants, informing device design and safety [73, 74]. Overall, large animal models are most useful when the research question requires human-like vessel caliber, operative technique, device deployment, or longitudinal translational assessment.

### Other Animal Models

Rabbit models bridge the anatomical gap between small and large animal models. Intermediate vessel size, ease of handling, and low perioperative mortality make rabbits particularly attractive for investigating vascular remodeling. Various AVF configurations have been successfully established in rabbits, including side-to-side and side-to-end anastomoses involving the common carotid artery, external jugular vein, abdominal aorta, and femoral vessels [61, 75–81] (Table 1). Notably, patency rates across studies are consistently high and often approach 100%, with durability demonstrated up to 50 weeks [76, 79]. Rabbit models have also enabled the study of CKD-related vascular changes through dietary induction of uremia [61]. Furthermore, larger vessel size allows for advanced imaging and interventional procedures not feasible in mice, while avoiding the substantial ethical and logistical burdens associated with larger species.

### Comparative Perspective

Taken together, animal models occupy distinct but complementary roles. While small animal models are frequently employed due to relatively lower cost, short reproductive cycles, and the availability of genetically modified strains [39–42], significant anatomical and physiological discrepancies—especially in vessel diameter, vascular wall composition, flow dynamics, WSS, and compliance—limit their capacity to replicate the clinical features of human AVF creation, maturation, and failure [23, 66]. In contrast, large animal models offer more anatomically and physiologically relevant platforms for translational research, but their use is constrained by substantial logistical, financial, and ethical burdens. These ethical considerations underpin the widespread implementation of the “Three Rs”—Replacement, Reduction, and Refinement—as guiding principles in animal research [82]. A tiered framework that uses small animals for mechanistic studies and large animals for translational and regulatory validation prior to clinical trials has therefore traditionally underpinned preclinical investigation [71, 72].

**Table 1:** Animal models in vascular access research

Animal species	Model method	Artery used	Vein used	Patency rates	Comments	Author name	Journal	Year	Reference
Mouse C57BL/J6 Adult mice	Side-to-End	Common carotid artery	External jugular vein	80–100% At 3 weeks, 33–58% patent at 4-6 weeks.	No concurrent CKD 20% Perioperative mortality	Castier, Y Castier, Y Juncos, JP Nath, KA Kwei, S Pike, D	Circ Res KI KI Am J Physiol Ren Physiol Am J Pathol Theor Biol Med Model	2005 2006 2008 2010 2004 2017	[23] [44] [47] [49] [120] [88]
Mouse Male C57BL/J6 10–15 Weeks of age	Side-to-End	Common carotid artery	External jugular vein	Not reported	Concurrent CKD with 5/6 subtotal nephrectomy 41.1–41.7% Perioperative mortality	Kang, K Kang, K	Am J Physiol Renal Physiol Am J Physiol Ren Physiol	2011 2015	[46] [45]
Mouse Male C57BL/6	Side-to-End	Common carotid artery	Dorsomedial branch of external jugular vein	88% At 7 days, 90% at 14 days, 50% at 28 days	No concurrent CKD 33-43% Perioperative mortality	Wong, CY Wong, CY Bezhaeva, T	J Vasc Surg Eur J Vasc Endovasc Surg FASEB J	2014 2015 2018	[121] [122] [123]
Mouse Male C57BL/6	Side-to-End	Common carotid artery	Dorsomedial branch of external jugular vein	86% Patency at 4 weeks.	Concurrent CKD with 5/6 subtotal nephrectomy Perioperative Mortality not reported	Shih, YC	Toxins	2020	[65]
Mouse Male FVB/NJ	End-to-End	Common carotid artery	External jugular vein	100% at 4 weeks.	No concurrent CKD 20% Perioperative mortality	Yang, B	J Vasc Interv Radiol	2009	[124]
Mouse Male C57BL/6	End-to-End	Common carotid artery	External jugular vein	Not reported	Concurrent CKD with L nephrectomy and ligation of blood supply to R upper pole Perioperative mortality not reported	Misra, S Janardhanan, R Janardhanan, R	J Vasc Interv Radiol Kidney Int J Vasc Interv Radiol	2010 2013 2016	[64] [53] [52]
Mouse Male C57BL/6	Side-to-Side	Aorta (infrarenal)	Inferior vena cava	75–80% Patency at 24 h, 80% patency at 21 days, 45-50% patency at 42 days	Aorta needle puncture into IVC No concurrent CKD 10–20% Perioperative mortality	Yamamoto, K Yamamoto, K Karram, T	J Vis Exp Am J Physiol Heart Circ Physiol J Investig Surg	2013 2013 2005	[125] [126] [127]
Mouse C57BL/6 Male	Side-to-Side	Aorta (infrarenal)	Inferior vena cava	Not reported	Aorta needle puncture into IVC Adenine diet-induced CKD Perioperative mortality not reported.	Liu, CT	Mol Med	2022	[48]
Mouse Male + Female C57BL/6	Side-to-Side	Aorta (infrarenal)	Inferior vena cava	26–65% Patency at 42 days	Aorta needle puncture into IVC No concurrent CKD. 12–18% Perioperative mortality	Kudze, T	JVS Vasc Sci	2020	[128]
Mouse Male C57BL/6	End-to-End	Common carotid artery	Internal jugular vein	78% Patency at 4 weeks	Concurrent CKD with 5/6 subtotal nephrectomy Perioperative mortality 22%	Juncos, JP Liang, A Guo, Q	J Am Soc Nephrol J Transl Med Am J Physiol Renal Physiol	2011 2013 2022	[59] [63] [50]
Mouse Male C57BL/6 J mice	Side-to-End	Common carotid artery	Internal jugular vein	50–80% Patency at 14 days, 20–50% patency at 28 days, 0–20% patency at 42 days	No concurrent CKD 11.1% Perioperative mortality.	Cui, J	J Am Soc Nephrol	2020	[129]
Rat Male Sprague–Dawley rats	Side-to-End	Femoral artery	Femoral vein	100% At 21 days	No concurrent CKD Perioperative mortality not reported	Northrup, H	Front Bioeng Biotechnol	2021	[87]
Rat Male Sprague–Dawley rats	Side-to-End	Femoral artery	Femoral vein	Not reported	Concurrent CKD with 5/6 subtotal nephrectomy Perioperative mortality not reported.	Croatt, AJ Juncos, JP Tsapenko, MV	Am J Pathol J Am Soc Nephrol Am J Physiol Ren Physiol	2010 2011 2012	[58] [59] [24]
Rat Female Wistar	Side-to-End	Femoral artery	Femoral vein	Not reported	No concurrent CKD Perioperative mortality not reported	Chan, CY	J Vasc Surg	2007	[130]
Rat Female Sprague–Dawley rats	Side-to-End	Femoral artery	Femoral vein	93–96% Patent at 12 weeks.	Adenine diet-induced CKD 0–12.5% Perioperative mortality	Langer, S Langer, S	KI J Vasc Access	2010 2011	[60] [61]
Rat Male Sprague–Dawley	Side-to-End	Common carotid artery	Internal jugular vein	100% At 8 weeks	No concurrent CKD 20% Perioperative mortality	Bai, H	Sci Trans Med	2020	[131]
Rat Male Sprague–Dawley	Side-to-Side	Femoral artery	Femoral vein	100% At 1 week	Proximal femoral artery ligated with silk suture 0% Perioperative mortality	McEnaney, RM Andraska, E	J Biol Methods Front Cardiovasc Med	2018 2022	[132] [133]
Rabbits Japanese White Male	Side-to-Side	Common carotid artery	External jugular vein	Not reported. 100% Up to 30 weeks	No concurrent CKD Not reported 0% Peri-operative mortality	Masuda, H Sho, E	Arterioscler Thromb Vasc Biol J Vasc Surg	1999 2004	[80] [81]
Rabbit New Zealand White	Side-to-Side	Common carotid artery	Common jugular vein	100% At 28 days	Adenine diet-induced CKD 0% Perioperative mortality	Li, Z	Int J Mol Med	2017	[62]
Rabbit New Zealand White	Side-to-Side	Abdominal aorta	Inferior vena cava	100% up to 50 weeks	No concurrent CKD 0% Perioperative mortality	Fallon, JT	Circ Res	1972	[77]
Rabbit New Zealand White	Side-to-End	Common carotid artery	External jugular vein	100% At 4 weeks	No concurrent CKD. 0% Peri-operative mortality	Ding, Y Tronc, F	AJNR Am J Neuroradiol Arterioscler Thromb Vasc Biol	2010 1996	[76] [82]
Rabbit New Zealand White	Side-to-Side	Femoral artery	Femoral vein	100% At 28 days	No concurrent CKD 0% Perioperative mortality	MackAskill, MG	Vasc Pharmacol	2015	[79]
Rabbit New Zealand White	Side-to-End with cuffed vein graft	Common carotid artery	External jugular vein	100% At 7 days	Contralateral external jugular vein used as graft and anastomosed to carotid artery No concurrent CKD 0% Perioperative mortality	Jiang, Z	Am J Physiol Heart Circ Physiol	2007	[78]
Dogs	Side-to-End	Distal caudal femoral artery	Lateral saphenous vein	100% (2/2 Dogs) at 56 days	No concurrent CKD 0% Perioperative mortality	Addin, CA	Vet Surg	2002	[74]
Dogs	Side-to-Side	Common carotid artery	External jugular vein						
Dogs	Side-to-Side	Brachial artery	Cephalic vein						
Dogs Greyhounds	Side-to-Side	Common carotid artery	External Jugular Vein	100% At all timepoints (20 days and 0, 3, 4, 5, 6, 10, 12 months)	No concurrent CKD 0% Perioperative mortality	Yang, L	Sci Rep	2024	[134]
Sheep	Side-to-Side	Common carotid artery	External jugular vein	100% At 20 months	No concurrent CKD 0% Perioperative mortality	Rogers, KM	Atherosclerosis	1985	[135]
Sheep Crossbred Border Leicester-Merino Wethers (castrated males)	Side -to-Side	Femoral artery	Femoral vein	Overall 95% (19/20 AVFs in 10 sheep): 100% (6/6) at 2 weeks, 87.5% (7/8) at 4 weeks, 100% (6/6) at 6 weeks	No concurrent CKD 0% Perioperative mortality	Varcoe, R	Eur J Vasc Endovasc Surg	2012	[68]
Sheep	Side-to-End	Brachial artery	Brachial vein	75% At 5 weeks	No concurrent CKD 33% Perioperative mortality reported (not related to AVF itself—1 aspiration pneumonia, 1 pre-existing liver abscess).	Florescu, MC	Semin Dial	2015	[73]
Sheep	Side-to-Side	Aorta (infrarenal)	Inferior vena cava	50% Patent at 2 weeks	No concurrent CKD 0% Perioperative mortality	Boudhghène, F	Circulation	1996	[136]
Pig Female Gottingen minipigs	Side-to-End	Common carotid artery	External jugular vein	100% At 4 weeks	No concurrent CKD 0% Perioperative mortality	Lovelands-Jones, CE	J Vasc Access	2014	[69]
Pig Domestic pig Female and castrated male juvenile	Side-to-End	Common carotid artery	External jugular vein	100% At 28 days.	Renal artery embolization 28 days prior to AVF creation 0% Perioperative mortality	Singh, AK	JASN	2021	[66]
Pig Female Mixed breed	Side-to-Side	External iliac artery	External iliac vein	80% At 64 days.	No concurrent CKD No perioperative mortality reported	Johnson, MS	J Vasc Interv Radiol	2001	[70]
Pig Yorkshire Cross domestic swine	Side-to-End	Femoral artery	Femoral vein	81% At 6 weeks	No concurrent CKD 0% Perioperative mortality	Wang, Y	Nephrol Dial Transplant	2008	[71]
Pig Landrace pigs	Side-to-End	Common carotid artery	Internal jugular vein	100% At 2 and 4 weeks, 50% at 8 weeks 100% At 2 weeks, 75% at 4 weeks, 50% at 12 weeks	No concurrent CKD 0% Perioperative mortality	Rotmans, JI Kritharis, EP	J Surg Res Biorheology	2003 2010	[137] [72]
Pigs Domestic pigs	Side-to-End	Common carotid artery	Internal jugular vein	57% At 29 days in higher endovascular radiation group, 87.5% at 56 days in lower endovascular radiation exposure group.	No concurrent CKD Perioperative mortality not reported	Rodriguez, V	J Vasc Interv Radiol	2002	[138]
Pigs Female Landrace pigs	Side-to-End	Femoral artery	Femoral vein	100% At 30 min following creation.	No concurrent CKD 0% Perioperative mortality	Barreiro, KA	Eur J Vasc Endovasc Surg	2019	[139]

### Fluid Dynamic Systems

CFD has become a central tool in vascular biology, enabling detailed simulation of the mechanical forces governing blood flow in AVFs, particularly in patient-specific configurations. CFD models can quantify the spatial and temporal distribution of WSS and characterize complex hemodynamic flow patterns at critical sites such as the anastomosis by incorporating vessel geometries and physiological waveform data. Accurate boundary conditions enhance the predictive capacity of these models, supporting both surgical planning and hypothesis-driven research [83].

CFD models have reliably predicted post-operative flow increases and provided quantitative insights into remodeling dynamics across diverse AVF types, including radiocephalic and brachiocephalic fistulas [84]. Under hemodialysis conditions, CFD has shown that needle placement modulates flow disturbances [85]. Turbulence persists from the anastomosis into the venous limb, with arterial needle extraction damping upstream flow and venous jet injection reintroducing turbulence. Metrics such as OSI and transverse WSS identify regions of endothelial stress associated with recirculation and neointimal hyperplasia [85]. Needle spacing had minimal effect on these stresses, suggesting limited scope for flow optimization through cannulation strategies alone. MRI-based CFD studies have also been used to map disturbed flow zones and correlate them with local vascular changes, supporting the hypotheses that flow heterogeneity drives localized neointimal hyperplasia [86, 87].

Beyond rigid-wall CFD, fluid–structure interaction (FSI) simulations are increasingly relevant because they couple blood flow to deformation of the vessel wall, enabling concurrent assessment of luminal hemodynamics and wall mechanics. This is particularly pertinent in AVFs, where abrupt changes in pressure, pulsatility, and compliance at the artery–vein anastomosis may generate not only abnormal luminal WSS but also altered intramural stress and wall displacement. In a compliant patient-specific AVF, FSI was used to quantify WSS, OSI, pressure, and arterial wall displacement, illustrating the additional biomechanical information gained when wall motion is incorporated [88]. More broadly, recent reviews of patient-specific vascular FSI emphasise that rigid-wall CFD alone cannot fully capture the interaction between blood flow and vascular wall mechanics [89]. This framework is also relevant to AVFs, where wall deformation and intramural stresses may shape maladaptive remodeling alongside luminal shear forces. FSI therefore expands computational AVF modelling beyond surface flow metrics to include the mechanical behavior of the vessel wall.

CFD simulations have also been used to evaluate the hemodynamic consequences of vascular interventions. Comparative modeling of AVFs before and after stent placement shows that flexible nitinol stents significantly reduce turbulent kinetic energy and cycle-to-cycle WSS variability, particularly at the heel of the anastomosis [90]. In models without stents, oscillatory flows emanating from the confluence of arterial inlets gives rise to greater turbulent activity and hemodynamic instability, while stented models funnel turbulent flow within the stent lumen and reduce recirculation zones [90, 91].

To validate CFD and FSI findings, macro-scale flow models have been used to directly study hemodynamic phenomena under physiologically relevant conditions. These systems replicate vascular geometries using silicone or compliant plastic tubing and are perfused with blood-mimicking fluids to achieve physiologic parameters. For example, upper extremity AVF models using elastic tubing and glycerine–water solutions to simulate blood viscosity allow detailed exploration of circulatory mechanics from the subclavian artery to the vein, including the introduction of pulsatile flow via heart simulation rigs [92]. Similar systems have demonstrated that extremes of WSS disrupt endothelial integrity and contribute to AVF non-maturation, supporting future work of vein preconditioning strategies [93].

Tomographic particle image velocimetry (PIV) in patient-specific benchtop models has revealed intricate 3D flow patterns such as zones of low velocity, recirculation, and elevated turbulent kinetic energy within the anastomosis and proximal vein [94]. In a patient-specific brachiocephalic AVF model, PIV showed persistent regions of flow separation and impingement at the anastomosis [95]. This was irrespective of pulsatility, with directional variability and high velocity fluctuations highest in regions of recirculation suggesting an elevated transverse and temporal WSS—both of which have been implicated in endothelial dysfunction and stenosis formation [95].

Across multiple anastomotic designs, regions of low shear within flow separation zones correspond to known sites of neointimal hyperplasia, typically located at the anastomotic heel and toe [96]. PIV not only offers high spatial and temporal resolution but has proven instrumental in validating CFD simulations. In one patient-specific AVF model, PIV data closely matched CFD predictions, and was used to inform mesh refinement strategies, thereby improving accuracy in the quantification of velocity and WSS distribution [97].

Beyond access design, macro-scale models have helped elucidate the impact of interventions such as interval ligation in ischemic steal syndrome, although technical limitations persist including the absence of venous return or fixed arterial inputs [98]. Ferumoxytol-enhanced magnetic resonance angiography now provides visualization of WSS and flow velocity, offering high-resolution validation of in vitro findings and accurate identification of stenosis-prone segments [99]. However, these macro-scale flow models inherently lack biological feedback, emphasizing the need for hybrid platforms that integrate mechanical stimuli with cellular responses to better capture mechanisms of maladaptive remodeling.

#### In Vitro Models

In vitro models provide controlled platforms to investigate discrete cellular and biomechanical components of AVF maturation and failure. The main value of these models lies in the ability to isolate specific stimuli and cell responses that are difficult to dissect in vivo. However, translational relevance depends on the extent to which multicellular interactions, extracellular matrix context, and physiologic flow conditions are reproduced.

#### Static and Simplified Cellular Models

Two-dimensional in vitro models provide simplified yet informative platforms to study cellular responses to biochemical and mechanical stimuli relevant to AVF maturation. These models typically employ single cell types such as ECs [100–102], VSMCs [101–105], or immune cells [102, 106]. Most studies have used primary cells, with a minority isolating cells directly from AVFs. ECs isolated from rabbit carotid arteries following AVF creation developed a persistent proliferative state after exposure to high shear stress even when cultured *ex vivo*, emphasizing the phenotypic changes after exposure to high flow [107]. These monoculture models are cost-effective, reproducible, and suitable for high-throughput assays, but lack the complex intercellular communication and extracellular matrix environment critical for AVF remodeling.

#### Multicellular and Patient-Derived Models

Recognition of the importance of cell-to-cell crosstalk has led to the development of co-culture and explant-based systems. Co-culture systems involving two or more cell types have allowed for characterisation of direct cellular interactions important in AVF maturation. For example, co-culture models have demonstrated that hypoxia-driven pathogenic pathways in AVFs rely on coordinated interactions between multiple vascular cell types [102]. Only co-culture of primary human fibroblasts, VSMCs, and ECs were capable of robustly inducing and secreting osteopontin under hypoxic conditions, through HIF-1α and NF-κB signaling, an observation that was not observed under monoculture conditions [102].

Recent studies have used primary tissue explant cultures directly derived from failed patient AVFs with intimal hyperplasia and compared these to normal veins [101, 108]. Primary tissue explant systems produce heterogeneous cultures that contain a physiological mixture of ECs, VSMCs, and fibroblasts mirroring the in vivo AVF wall [101, 108]. Analyses of these patient-derived AVF cell populations has identified significant upregulation of specific microRNAs associated with increased proliferation, migration, and inflammatory cytokine secretion—hallmarks of intimal hyperplasia [108]. Pharmacological targeting, such as PPAR-γ inhibition, increases both cell proliferation and gene expression of inflammatory mediators and remodeling-related transcripts in these mixed-cell cultures [101], supporting the translational potential of targeting multicellular signaling pathways involved in AVF failure [101]. Three-dimensional (3D) culture systems more closely replicate in vivo environments [109–112], but have not been widely utilized in AVF research to date.

#### Flow-Based Culture Systems

Flow-based culture systems attempt to bridge the gap between reductionist cell culture and hemodynamically realistic modeling. Microfluidic systems, often referred to as organ-on-a-chip or vessel-on-a-chip platforms, represent a cutting-edge advancement in vascular biology research [113]. These devices use microfabricated channels lined with ECs, sometimes in conjunction with VSMCs or other stromal components, enabling real-time monitoring of vascular responses under precisely controlled hemodynamic and biochemical conditions [114]. Despite clear benefits over static in vitro systems, their use in AVF research has largely been limited to indirect examination of mechanisms relevant to AVFs. At the endothelial surface, a 2D microfluidic system incorporating ridge-shaped obstacles examined the effect of disturbed flow on primary human aortic endothelial cells, HAECs [115]. HAECs exhibited cytoskeletal remodeling characterized by perpendicular alignment of actin stress fibers relative to the direction of flow, increased nuclear circularity, and reduced nuclear area under disturbed flow conditions. In parallel, a 3D cardiac tissue chip that simulated volume overload conditions analogous to those occurring after AVF creation measured physiologically relevant outputs such as collagen deposition, gene expression changes related to fibrosis and oxidative stress, and mechanical strain (~ 9% under normal loading vs. ~ 18% under volume overload) [116]. Despite their physiologic advantages over static systems, broader adoption has been limited by technical challenges in recreating complex AVF-like geometries, the need for specialized micro-engineering capabilities, and sensitivity of microfluidic channels to bubble formation [117].

Macrofluidic platforms represent a complementary approach. Prior macrofluidic models have integrated perfusion circuits with blood-mimicking fluids enabling quantitative WSS assessment across vein diameters under physiological conditions [94, 118]. However, these models did not include cells, precluding biological readouts of signaling or cellular responses. To date, only a single 3D in vitro model has incorporated a cellular monolayer to investigate the effect of hemodialysis-induced turbulent flow on the endothelium: in this system, bovine aortic endothelial cells cultured on compliant silicone tubes coated with fibronectin were subjected to either steady laminar flow or combined with a physiologically relevant turbulent jet produced by a dialysis needle [119]. The model revealed that turbulent needle flow led to significant endothelial cell loss, disrupted endothelial alignment, and markedly decreased nitric oxide production—none of which occurred with laminar flow alone [119].

#### Comparative Perspective

Overall, in vitro AVF models show a clear progression from simplified monoculture systems, which are best suited for mechanistic reductionism and screening, toward multicellular, patient-derived, and flow-based systems that offer increasing physiologic relevance. The major limitation of these models remains the difficulty of integrating hemodynamic realism with multicellular biological function, particularly using clinically relevant flow conditions and cells that accurately simulate in vivo physiological responses. The greatest promise lies in combining computational flow modeling with engineered vascular constructs. While macrofluidic AVF platforms are under active development [120], truly integrated human-relevant systems remain limited.

## Conclusions

AVF maturation is not determined by blood flow alone, but by the interaction between local hemodynamics, vessel wall adaptation, and the systemic uremic environment. Across clinical, animal, and computational studies, successful maturation has been associated with coordinated outward remodeling and relative normalization of the early postoperative hemodynamic insult, whereas failure is repeatedly linked to persistent disturbed flow, oxidative stress, inflammation, impaired re-endothelialization, and excessive neointimal hyperplasia (Fig. 2).

Existing models capture important but isolated elements of this complexity and are complementary rather than interchangeable. Small animal models have played important roles in identifying causal molecular pathways, including nitric oxide signaling, MMP activity, oxidative stress, inflammatory macrophage recruitment, endothelial-to-mesenchymal transition, and the deleterious effects of CKD on vascular adaptation. Large animal models have provided stronger translational fidelity by reproducing human-scale geometry, surgical workflows, and the spatial relationship between disturbed flow and intimal hyperplasia. CFD has clarified where disturbed shear, recirculation, and turbulence arise within the AVF, and emerging FSI studies indicate that vessel compliance, wall deformation, and possibly flow-induced wall vibration may carry mechanobiological information beyond luminal WSS alone. In vitro and flow-based culture systems, while less anatomically complete, offer the most controlled platforms for dissecting human-cell responses to defined mechanical and biochemical stimuli.

The major gap in the field is not the absence of models, but the absence of integrated models that combine human-relevant geometry, realistic wall mechanics, uremic biochemistry, and multicellular biological readouts. No single current platform reproduces all of these features simultaneously. For that reason, the most informative near-term strategy is a multimodal pipeline in which animal models identify candidate mechanisms, patient-specific CFD and FSI localize adverse mechanical environments, and endothelialized flow platforms test how human vascular cells respond to those environments under uremic conditions before large-animal validation of promising interventions.

Future progress in AVF research will depend less on developing one “ideal” model than on deliberately linking complementary models across scales. Such an approach is more likely to yield mechanistic biomarkers of maturation failure, identify tractable therapeutic targets, and support the rational design of interventions aimed at improving AVF patency, durability, and clinical usability.

## Data Availability

All data used in this narrative review are publicly available from previously published studies. Citations and references for the included studies are provided within the manuscript and supplementary materials. Not applicable.

## References

[1] Chadban, S. J., et al. Prevalence of kidney damage in Australian adults: the AusDiab kidney study. *J. Am. Soc. Nephrol.* 14(7 Suppl 2):S131–S138, 2003. 12819318 10.1097/01.asn.0000070152.11927.4a

[2] Hallan, S. I., et al. International comparison of the relationship of chronic kidney disease prevalence and ESRD risk. *J. Am. Soc. Nephrol.* 17(8):2275–2284, 2006. 16790511 10.1681/ASN.2005121273

[3] Coresh, J., et al. Prevalence of chronic kidney disease and decreased kidney function in the adult US population: Third National Health and Nutrition Examination Survey. *Am. J. Kidney Dis.* 41(1):1–12, 2003. 12500213 10.1053/ajkd.2003.50007

[4] GBD Chronic Kidney Disease Collaboration. Global, regional, and national burden of chronic kidney disease, 1990–2017: a systematic analysis for the Global Burden of Disease Study 2017. *Lancet*. 395(10225):709–733, 2020. 32061315 10.1016/S0140-6736(20)30045-3 PMC7049905

[5] World Health Organisation. Global Health Estimates 2019: Deaths by Cause, Age, Sex by Country and by Region, 2000–2019. WHO, 2019. https://www.who.int/news-room/fact-sheets/detail/the-top-10-causes-of-death.

[6] Liyanage, T., et al. Worldwide access to treatment for end-stage kidney disease: a systematic review. *Lancet*. 385(9981):1975–1982, 2015. 25777665 10.1016/S0140-6736(14)61601-9

[7] Lok, C. E., et al. KDOQI clinical practice guideline for vascular access: 2019 update. *Am. J. Kidney Dis.* 75(4 Suppl 2):S1–S164, 2020. 32778223 10.1053/j.ajkd.2019.12.001

[8] Almasri, J., et al. Outcomes of vascular access for hemodialysis: a systematic review and meta-analysis. *J. Vasc. Surg.* 64(1):236–243, 2016. 27345510 10.1016/j.jvs.2016.01.053

[9] Shah, N. A., et al. Predicting high-flow arteriovenous fistulas and cardiac outcomes in hemodialysis patients. *J. Vasc. Surg.* 81(3):751-758.e8, 2025. 39631475 10.1016/j.jvs.2024.11.028

[10] Shah, N. A., et al. A change of heart: the effect of high flow arteriovenous fistulas on cardiovascular outcomes—a systematic review and synthesis without meta-analysis (SWiM). *BMC Nephrol.* 26(1):394, 2025. 40676508 10.1186/s12882-025-04324-8 PMC12273289

[11] Kruger-Genge, A., et al. Vascular endothelial cell biology: an update. *Int. J. Mol. Sci.* 20(18):4411, 2019. 31500313 10.3390/ijms20184411 PMC6769656

[12] Nakajima, H., and N. Mochizuki. Flow pattern-dependent endothelial cell responses through transcriptional regulation. *Cell Cycle*. 16(20):1893–1901, 2017. 28820314 10.1080/15384101.2017.1364324 PMC5638382

[13] Zhou, J., Y. S. Li, and S. Chien. Shear stress-initiated signaling and its regulation of endothelial function. *Arterioscler. Thromb. Vasc. Biol.* 34(10):2191–2198, 2014. 24876354 10.1161/ATVBAHA.114.303422 PMC4169328

[14] Pan, S. Molecular mechanisms responsible for the atheroprotective effects of laminar shear stress. *Antioxid. Redox Signal.* 11(7):1669–1682, 2009. 19309258 10.1089/ars.2009.2487 PMC2842586

[15] Vila Cuenca, M., P. L. Hordijk, and M. G. Vervloet. Most exposed: the endothelium in chronic kidney disease. *Nephrol. Dial. Transplant.* 35(9):1478–1487, 2020. 31071222 10.1093/ndt/gfz055 PMC7473805

[16] Harlacher, E., et al. Impact of uremic toxins on endothelial dysfunction in chronic kidney disease: a systematic review. *Int. J. Mol. Sci.* 23(1):531, 2022. 35008960 10.3390/ijms23010531 PMC8745705

[17] Corpataux, J. M., et al. Low-pressure environment and remodelling of the forearm vein in Brescia-Cimino haemodialysis access. *Nephrol. Dial. Transplant.* 17(6):1057–1062, 2002. 12032197 10.1093/ndt/17.6.1057

[18] Miller, V. M., and J. C. Burnett Jr. Modulation of NO and endothelin by chronic increases in blood flow in canine femoral arteries. *Am. J. Physiol.* 263(1 Pt 2):H103–H108, 1992. 1636749 10.1152/ajpheart.1992.263.1.H103

[19] Tronc, F., et al. Role of NO in flow-induced remodeling of the rabbit common carotid artery. *Arterioscler. Thromb. Vasc. Biol.* 16(10):1256–1262, 1996. 8857922 10.1161/01.atv.16.10.1256

[20] Tohda, K., et al. Difference in dilatation between endothelium-preserved and -desquamated segments in the flow-loaded rat common carotid artery. *Arterioscler. Thromb.* 12(4):519–528, 1992. 1558839 10.1161/01.atv.12.4.519

[21] Kamiya, A., and T. Togawa. Adaptive regulation of wall shear stress to flow change in the canine carotid artery. *Am. J. Physiol.* 239(1):H14–H21, 1980. 7396013 10.1152/ajpheart.1980.239.1.H14

[22] Ben Driss, A., et al. Arterial expansive remodeling induced by high flow rates. *Am. J. Physiol.* 272(2 Pt 2):H851–H858, 1997. 9124448 10.1152/ajpheart.1997.272.2.H851

[23] Castier, Y., et al. p47phox-dependent NADPH oxidase regulates flow-induced vascular remodeling. *Circ. Res.* 97(6):533–540, 2005. 16109921 10.1161/01.RES.0000181759.63239.21

[24] Tsapenko, M. V., et al. Increased production of superoxide anion contributes to dysfunction of the arteriovenous fistula. *Am. J. Physiol. Ren. Physiol.* 303(12):F1601–F1607, 2012. 10.1152/ajprenal.00449.2012 PMC3532470 22993073

[25] Dammers, R., et al. The effect of flow changes on the arterial system proximal to an arteriovenous fistula for hemodialysis. *Ultrasound Med. Biol.* 31(10):1327–1333, 2005. 16223635 10.1016/j.ultrasmedbio.2005.03.017

[26] Wong, V., et al. Factors associated with early failure of arteriovenous fistulae for haemodialysis access. *Eur. J. Vasc. Endovasc. Surg.* 12(2):207–213, 1996. 8760984 10.1016/s1078-5884(96)80108-0

[27] Nath, K. A., et al. Increased venous proinflammatory gene expression and intimal hyperplasia in an aorto-caval fistula model in the rat. *Am. J. Pathol.* 162(6):2079–2090, 2003. 12759262 10.1016/S0002-9440(10)64339-8 PMC1868137

[28] He, Y., et al. Association of shear stress with subsequent lumen remodeling in hemodialysis arteriovenous fistulas. *Clin. J. Am. Soc. Nephrol.* 18(1):72–83, 2023. 36446600 10.2215/CJN.04630422 PMC10101625

[29] Gorecka, J., et al. Molecular targets for improving arteriovenous fistula maturation and patency. *Vasc. Investig. Ther.* 2(2):33–41, 2019. 31608322 10.4103/VIT.VIT_9_19 PMC6788624

[30] Hashimoto, T., et al. Stimulation of caveolin-1 signaling improves arteriovenous fistula patency. *Arterioscler. Thromb. Vasc. Biol.* 39(4):754–764, 2019. 30786746 10.1161/ATVBAHA.119.312417 PMC6436985

[31] Zhang, W., et al. Endothelial TGF-beta signaling regulates endothelial–mesenchymal transition during arteriovenous fistula remodeling in mice with chronic kidney disease. *Arterioscler. Thromb. Vasc. Biol.* 44(12):2509–2526, 2024. 39297205 10.1161/ATVBAHA.124.320933 PMC11593991

[32] Xu, Y., et al. Inhibition of endothelial-to-mesenchymal transition in a large animal preclinical arteriovenous fistula model leads to improved remodelling and reduced stenosis. *Cardiovasc. Res.* 120(14):1768–1779, 2024. 39056563 10.1093/cvr/cvae157 PMC11587554

[33] Weiss, M. F., V. Scivittaro, and J. M. Anderson. Oxidative stress and increased expression of growth factors in lesions of failed hemodialysis access. *Am. J. Kidney Dis.* 37(5):970–980, 2001. 11325679 10.1016/s0272-6386(05)80013-7

[34] Chang, C. J., et al. Thrombosed arteriovenous fistula for hemodialysis access is characterized by a marked inflammatory activity. *Kidney Int.* 68(3):1312–1319, 2005. 16105066 10.1111/j.1523-1755.2005.00529.x

[35] Shih, Y. C., et al. MMP-9 deletion attenuates arteriovenous fistula neointima through reduced perioperative vascular inflammation. *Int. J. Mol. Sci.* 22(11):5448, 2021. 34064140 10.3390/ijms22115448 PMC8196691

[36] Correia, A. L., et al. Predictive factors for arteriovenous fistula maturation: a prospective study. *Hemodial. Int.* 29(1):24–30, 2025. 39690913 10.1111/hdi.13193

[37] Martinez, L., et al. Systemic profile of cytokines in arteriovenous fistula patients and their associations with maturation failure. *Kidney360*. 3(4):677–686, 2022. 35721613 10.34067/KID.0006022021 PMC9136910

[38] Nguyen, M., F. G. Thankam, and D. K. Agrawal. Sterile inflammation in the pathogenesis of maturation failure of arteriovenous fistula. *J. Mol. Med. (Berl.)*. 99(6):729–741, 2021. 33666676 10.1007/s00109-021-02056-4

[39] Simmons, D. The use of animal models in studying genetic disease: transgenesis and induced mutation. *Nat. Educ.* 1(1):70, 2008.

[40] Gibbs, R. A., et al. Genome sequence of the Brown Norway rat yields insights into mammalian evolution. *Nature*. 428(6982):493–521, 2004. 15057822 10.1038/nature02426

[41] Mouse Genome Sequencing Consortium. Initial sequencing and comparative analysis of the mouse genome. *Nature*. 420(6915):520–562, 2002. 12466850 10.1038/nature01262

[42] Bryda, E. C. The Mighty Mouse: the impact of rodents on advances in biomedical research. *Mo Med.* 110(3):207–211, 2013. 23829104 PMC3987984

[43] Castier, Y., et al. Characterization of neointima lesions associated with arteriovenous fistulas in a mouse model. *Kidney Int.* 70(2):315–320, 2006. 16760906 10.1038/sj.ki.5001569

[44] Kang, L., et al. A new model of an arteriovenous fistula in chronic kidney disease in the mouse: beneficial effects of upregulated heme oxygenase-1. *Am. J. Physiol. Ren. Physiol.* 310(6):F466–F476, 2016. 10.1152/ajprenal.00288.2015 PMC4796269 26672617

[45] Kang, L., et al. Regional and systemic hemodynamic responses following the creation of a murine arteriovenous fistula. *Am. J. Physiol. Ren. Physiol.* 301(4):F845–F851, 2011. 10.1152/ajprenal.00311.2011 PMC3191805 21697243

[46] Juncos, J. P., et al. Genetic deficiency of heme oxygenase-1 impairs functionality and form of an arteriovenous fistula in the mouse. *Kidney Int.* 74(1):47–51, 2008. 18368029 10.1038/ki.2008.110 PMC2914571

[47] Liu, C. T., et al. Inhibition of beta-catenin signaling attenuates arteriovenous fistula thickening in mice by suppressing myofibroblasts. *Mol. Med.* 28(1):7, 2022. 35062862 10.1186/s10020-022-00436-1 PMC8783463

[48] Nath, K. A., et al. ss-Catenin is markedly induced in a murine model of an arteriovenous fistula: the effect of metalloproteinase inhibition. *Am. J. Physiol. Ren. Physiol.* 299(6):F1270–F1277, 2010. 10.1152/ajprenal.00488.2010 PMC3006312 20881035

[49] Guo, Q., et al. Temporal regulation of notch activation improves arteriovenous fistula maturation. *J. Transl. Med.* 20(1):543, 2022. 36419038 10.1186/s12967-022-03727-7 PMC9682688

[50] Misra, S., et al. Anti human CX3CR1 VHH molecule attenuates venous neointimal hyperplasia of arteriovenous fistula in mouse model. *J. Am. Soc. Nephrol.* 32(7):1630–1648, 2021. 33893223 10.1681/ASN.2020101458 PMC8425661

[51] Janardhanan, R., et al. The role of repeat administration of adventitial delivery of lentivirus-shRNA-VEGF-A in arteriovenous fistula to prevent venous stenosis formation. *J. Vasc. Interv. Radiol.* 27(4):576–583, 2016. 26948326 10.1016/j.jvir.2015.12.751 PMC4808445

[52] Janardhanan, R., et al. Simvastatin reduces venous stenosis formation in a murine hemodialysis vascular access model. *Kidney Int.* 84(2):338–352, 2013. 23636169 10.1038/ki.2013.112 PMC3731558

[53] Rong, D., et al. The study of rapamycin nanofibrous membrane for preventing arteriovenous fistula stenosis. *Mater. Des.*245:113297, 2024.

[54] Somarathna, M., et al. Nitric oxide releasing nanomatrix gel treatment inhibits venous intimal hyperplasia and improves vascular remodeling in a rodent arteriovenous fistula. *Biomaterials*.280:121254, 2022. 34836683 10.1016/j.biomaterials.2021.121254 PMC8724452

[55] Nieves Torres, E. C., et al. Adventitial delivery of lentivirus-shRNA-ADAMTS-1 reduces venous stenosis formation in arteriovenous fistula. *PLoS ONE*.9(4):e94510, 2014. 24732590 10.1371/journal.pone.0094510 PMC3986087

[56] Cai, C., et al. Therapeutic effect of adipose derived mesenchymal stem cell transplantation in reducing restenosis in a murine angioplasty model. *J. Am. Soc. Nephrol.* 31(8):1781–1795, 2020. 32587073 10.1681/ASN.2019101042 PMC7460892

[57] Croatt, A. J., et al. Characterization of a model of an arteriovenous fistula in the rat: the effect of L-NAME. *Am. J. Pathol.* 176(5):2530–2541, 2010. 20363917 10.2353/ajpath.2010.090649 PMC2861117

[58] Juncos, J. P., et al. MCP-1 contributes to arteriovenous fistula failure. *J. Am. Soc. Nephrol.* 22(1):43–48, 2011. 21115617 10.1681/ASN.2010040373 PMC3014033

[59] Langer, S., et al. Chronic kidney disease aggravates arteriovenous fistula damage in rats. *Kidney Int.* 78(12):1312–1321, 2010. 20881937 10.1038/ki.2010.353

[60] Langer, S., et al. Cardiovascular remodeling during arteriovenous fistula maturation in a rodent uremia model. *J. Vasc. Access*. 12(3):215–223, 2011. 21104672 10.5301/JVA.2010.6066

[61] Li, Z., et al. Hyperbaric oxygen inhibits venous neointimal hyperplasia following arteriovenous fistulization. *Int. J. Mol. Med.* 39(5):1299–1306, 2017. 28393184 10.3892/ijmm.2017.2948

[62] Liang, A., et al. Chronic kidney disease accelerates endothelial barrier dysfunction in a mouse model of an arteriovenous fistula. *Am. J. Physiol. Ren. Physiol.* 304(12):F1413–F1420, 2013. 10.1152/ajprenal.00585.2012 PMC3680675 23576636

[63] Misra, S., et al. Increased expression of HIF-1alpha, VEGF-A and its receptors, MMP-2, TIMP-1, and ADAMTS-1 at the venous stenosis of arteriovenous fistula in a mouse model with renal insufficiency. *J. Vasc. Interv. Radiol.* 21(8):1255–1261, 2010. 20598569 10.1016/j.jvir.2010.02.043 PMC2910198

[64] Shih, Y. C., et al. Oral charcoal adsorbents attenuate neointima formation of arteriovenous fistulas. *Toxins (Basel)*. 12(4):237, 2020. 32276394 10.3390/toxins12040237 PMC7232464

[65] Singh, A. K., et al. 1Alpha, 25-dihydroxyvitamin D(3) encapsulated in nanoparticles prevents venous neointimal hyperplasia and stenosis in porcine arteriovenous fistulas. *J. Am. Soc. Nephrol.* 32(4):866–885, 2021. 33627344 10.1681/ASN.2020060832 PMC8017547

[66] Krishnamoorthy, M. K., et al. Hemodynamic wall shear stress profiles influence the magnitude and pattern of stenosis in a pig AV fistula. *Kidney Int.* 74(11):1410–1419, 2008. 18818686 10.1038/ki.2008.379

[67] Varcoe, R. L., et al. An arteriovenous fistula model of intimal hyperplasia for evaluation of a nitinol U-Clip anastomosis. *Eur. J. Vasc. Endovasc. Surg.* 43(2):224–231, 2012. 22104322 10.1016/j.ejvs.2011.11.002

[68] Loveland-Jones, C. E., et al. A new model of arteriovenous fistula to study hemodialysis access complications. *J. Vasc. Access*. 15(5):351–357, 2014. 24811594 10.5301/jva.5000222

[69] Johnson, M. S., et al. The porcine hemodialysis access model. *J. Vasc. Interv. Radiol.* 12(8):969–977, 2001. 11487678 10.1016/s1051-0443(07)61578-4

[70] Wang, Y., et al. Venous stenosis in a pig arteriovenous fistula model—anatomy, mechanisms and cellular phenotypes. *Nephrol. Dial. Transplant.* 23(2):525–533, 2008. 18037619 10.1093/ndt/gfm547

[71] Kritharis, E. P., et al. Biomechanical, morphological and zero-stress state characterization of jugular vein remodeling in arteriovenous fistulas for hemodialysis. *Biorheology*. 47(5–6):297–319, 2010. 21403383 10.3233/BIR-2011-0578

[72] Florescu, M. C., et al. Sheep model of hemodialysis arteriovenous fistula using superficial veins. *Semin. Dial.* 28(6):687–691, 2015. 26189959 10.1111/sdi.12407

[73] Adin, C. A., et al. Evaluation of three peripheral arteriovenous fistulas for hemodialysis access in dogs. *Vet. Surg.* 31(5):405–411, 2002. 12209410 10.1053/jvet.2002.34663

[74] Yang, L., et al. Electrospun silk fibroin/fibrin vascular scaffold with superior mechanical properties and biocompatibility for applications in tissue engineering. *Sci. Rep.* 14(1):3942, 2024. 38365964 10.1038/s41598-024-54638-0 PMC10873321

[75] Ding, Y., et al. Creation of large elastase-induced aneurysms: presurgical arterial remodeling using arteriovenous fistulas. *Am. J. Neuroradiol.* 31(10):1935–1937, 2010. 20634302 10.3174/ajnr.A2205 PMC2974950

[76] Fallon, J. T., and W. E. Stehbens. Venous endothelium of experimental arteriovenous fistulas in rabbits. *Circ. Res.* 31(4):546–556, 1972. 4116427 10.1161/01.res.31.4.546

[77] Jiang, Z., et al. TGF-beta- and CTGF-mediated fibroblast recruitment influences early outward vein graft remodeling. *Am. J. Physiol. Heart Circ. Physiol.* 293(1):H482–H488, 2007. 17369455 10.1152/ajpheart.01372.2006

[78] MacAskill, M. G., et al. Improving arteriovenous fistula patency: transdermal delivery of diclofenac reduces cannulation-dependent neointimal hyperplasia via AMPK activation. *Vasc. Pharmacol.* 71:108–115, 2015. 10.1016/j.vph.2015.02.012 PMC4534710 25866325

[79] Masuda, H., et al. Adaptive remodeling of internal elastic lamina and endothelial lining during flow-induced arterial enlargement. *Arterioscler. Thromb. Vasc. Biol.* 19(10):2298–2307, 1999. 10521357 10.1161/01.atv.19.10.2298

[80] Sho, E., et al. Arterial enlargement, tortuosity, and intimal thickening in response to sequential exposure to high and low wall shear stress. *J. Vasc. Surg.* 39(3):601–612, 2004. 14981455 10.1016/j.jvs.2003.10.058

[81] Tronc, F., et al. Role of matrix metalloproteinases in blood flow-induced arterial enlargement: interaction with NO. *Arterioscler. Thromb. Vasc. Biol.* 20(12):E120–E126, 2000. 11116076 10.1161/01.atv.20.12.e120

[82] Russell, W. M. S., and R. L. Burch. The principles of humane experimental technique. *Med. J. Aust.* 1(13):500, 1960.

[83] Ng, O., et al. The effect of assumed boundary conditions on the accuracy of patient-specific CFD arteriovenous fistula model. *Comput. Methods Biomech. Biomed. Eng. Imaging Vis.* 11(1):31–43, 2023.

[84] Caroli, A., et al. Validation of a patient-specific hemodynamic computational model for surgical planning of vascular access in hemodialysis patients. *Kidney Int.* 84(6):1237–1245, 2013. 23715122 10.1038/ki.2013.188

[85] Fulker, D., B. Ene-Iordache, and T. Barber. High-resolution computational fluid dynamic simulation of haemodialysis cannulation in a patient-specific arteriovenous fistula. *J. Biomech. Eng.* 2018. 10.1115/1.4038289. 29080304 10.1115/1.4038289

[86] Northrup, H., et al. Analysis of geometric and hemodynamic profiles in rat arteriovenous fistula following PDE5A inhibition. *Front. Bioeng. Biotechnol.*9:779043, 2021. 34926425 10.3389/fbioe.2021.779043 PMC8675087

[87] Pike, D., et al. High resolution hemodynamic profiling of murine arteriovenous fistula using magnetic resonance imaging and computational fluid dynamics. *Theor. Biol. Med. Model.* 14(1):5, 2017. 28320412 10.1186/s12976-017-0053-x PMC5360029

[88] Decorato, I., et al. Numerical simulation of the fluid structure interactions in a compliant patient-specific arteriovenous fistula. *Int. J. Numer. Method Biomed. Eng.* 30(2):143–159, 2014. 24493402 10.1002/cnm.2595

[89] Schoenborn, S., et al. Fluid–structure interaction within models of patient-specific arteries: computational simulations and experimental validations. *IEEE Rev. Biomed. Eng.* 17:280–296, 2024. 36260570 10.1109/RBME.2022.3215678

[90] Gunasekera, S., et al. Mitigation of the turbulence within an arteriovenous fistula with a stent implantation. *Phys. Rev. Fluids*.7(12):123101, 2022.

[91] Gunasekera, S., et al. Stenosis to stented: decrease in flow disturbances following stent implantation of a diseased arteriovenous fistula. *Biomech. Model. Mechanobiol.* 23(2):453–468, 2024. 38063956 10.1007/s10237-023-01784-5

[92] Varble, N., et al. In vitro hemodynamic model of the arm arteriovenous circulation to study hemodynamics of native arteriovenous fistula and the distal revascularization and interval ligation procedure. *J. Vasc. Surg.* 59(5):1410–1417, 2014. 23845661 10.1016/j.jvs.2013.04.055

[93] Loree, H. M., II., et al. In vitro study of a medical device to enhance arteriovenous fistula eligibility and maturation. *ASAIO J.* 61(4):480–486, 2015. 26120958 10.1097/MAT.0000000000000240

[94] Gunasekera, S., et al. Tomographic PIV analysis of physiological flow conditions in a patient-specific arteriovenous fistula. *Exp. Fluids*. 61(12):253, 2020.

[95] Alam, N., M. Walsh, and D. Newport. Experimental evaluation of a patient specific Brachio-Cephalic Arterio Venous Fistula (AVF): velocity flow conditions under steady and pulsatile waveforms. *Med. Eng. Phys.*106:103834, 2022. 35926957 10.1016/j.medengphy.2022.103834

[96] Heise, M., et al. Flow pattern and shear stress distribution of distal end-to-side anastomoses. A comparison of the instantaneous velocity fields obtained by particle image velocimetry. *J. Biomech.* 37(7):1043–1051, 2004. 15165874 10.1016/j.jbiomech.2003.11.030

[97] Kharboutly, Z., et al. Numerical and experimental study of blood flow through a patient-specific arteriovenous fistula used for hemodialysis. *Med. Eng. Phys.* 32(2):111–118, 2010. 19962337 10.1016/j.medengphy.2009.10.013

[98] Zanow, J., et al. Experimental study of hemodynamics in procedures to treat access-related ischemia. *J. Vasc. Surg.* 48(6):1559–1565, 2008. 18771888 10.1016/j.jvs.2008.06.040

[99] Hyde-Linaker, G., et al. Patient-specific computational haemodynamics associated with the surgical creation of an arteriovenous fistula. *Med. Eng. Phys.*105:103814, 2022. 35781379 10.1016/j.medengphy.2022.103814

[100] Xu, M., et al. Increased expression of angiogenic factors in cultured human brain arteriovenous malformation endothelial cells. *Cell Biochem. Biophys.* 70(1):443–447, 2014. 24771337 10.1007/s12013-014-9937-0

[101] Ciavarella, C., et al. The PPAR-gamma agonist pioglitazone modulates proliferation and migration in HUVEC, HAOSMC and human arteriovenous fistula-derived cells. *Int. J. Mol. Sci.* 24(5):4424, 2023. 36901853 10.3390/ijms24054424 PMC10003103

[102] Sadaghianloo, N., et al. Co-culture of human fibroblasts, smooth muscle and endothelial cells promotes osteopontin induction in hypoxia. *J. Cell Mol. Med.* 24(5):2931–2941, 2020. 32032472 10.1111/jcmm.14905 PMC7077551

[103] Stegemann, J. P., and R. M. Nerem. Altered response of vascular smooth muscle cells to exogenous biochemical stimulation in two- and three-dimensional culture. *Exp. Cell Res.* 283(2):146–155, 2003. 12581735 10.1016/s0014-4827(02)00041-1

[104] Hu, K., et al. Egr-1 promotes the proliferation and migration of vascular smooth muscle cells by transcriptionally activating Egr-2 in arteriovenous fistulas. *Int. J. Mol. Med.* 56(3):127, 2025. 40576118 10.3892/ijmm.2025.5568 PMC12215247

[105] Liu, C. T., et al. Parathyroid hormone induces transition of myofibroblasts in arteriovenous fistula and increases maturation failure. *Endocrinology*. 2021. 10.1210/endocr/bqab044. 33640969 10.1210/endocr/bqab044

[106] Liang, M., et al. Notch signaling in bone marrow-derived FSP-1 cells initiates neointima formation in arteriovenous fistulas. *Kidney Int.* 95(6):1347–1358, 2019. 30799025 10.1016/j.kint.2018.11.027 PMC6763204

[107] Yamauchi, M., et al. Normalization of high-flow or removal of flow cannot stop high-flow induced endothelial proliferation. *Biomed. Res.* 26(1):21–28, 2005. 15806980 10.2220/biomedres.26.21

[108] Ciavarella, C., et al. A distinct miRNA profile in intimal hyperplasia of failed arteriovenous fistulas reveals key pathogenic pathways. *Biomolecules*. 15(8):1064, 2025. 40867509 10.3390/biom15081064 PMC12383664

[109] Azar, J., et al. The use of stem cell-derived organoids in disease modeling: an update. *Int. J. Mol. Sci.* 22(14):7667, 2021. 34299287 10.3390/ijms22147667 PMC8303386

[110] Kennedy, K. M., A. Bhaw-Luximon, and D. Jhurry. Cell–matrix mechanical interaction in electrospun polymeric scaffolds for tissue engineering: Implications for scaffold design and performance. *Acta Biomater.* 50:41–55, 2017. 28011142 10.1016/j.actbio.2016.12.034

[111] Langhans, S. A. Three-dimensional in vitro cell culture models in drug discovery and drug repositioning. *Front. Pharmacol.* 9:6, 2018. 29410625 10.3389/fphar.2018.00006 PMC5787088

[112] Jensen, C., and Y. Teng. Is it time to start transitioning from 2D to 3D cell culture? *Front. Mol. Biosci.* 7:33, 2020. 32211418 10.3389/fmolb.2020.00033 PMC7067892

[113] Gurkan, U. A., et al. Next generation microfluidics: fulfilling the promise of lab-on-a-chip technologies. *Lab. Chip*. 24(7):1867–1874, 2024. 38487919 10.1039/d3lc00796k PMC10964744

[114] Hu, C., et al. Microfluidic technologies for vasculature biomimicry. *Analyst*. 144(15):4461–4471, 2019. 31162494 10.1039/c9an00421a

[115] Tovar-Lopez, F., et al. A microfluidic system for studying the effects of disturbed flow on endothelial cells. *Front. Bioeng. Biotechnol.* 7:81, 2019. 31111027 10.3389/fbioe.2019.00081 PMC6499196

[116] Waldrop, T. I., et al. Biomimetic cardiac tissue chip and murine arteriovenous fistula models for recapitulating clinically relevant cardiac remodeling under volume overload conditions. *Front. Bioeng. Biotechnol.* 11:1101622, 2023. 36873372 10.3389/fbioe.2023.1101622 PMC9978753

[117] Bhatia, S. N., and D. E. Ingber. Microfluidic organs-on-chips. *Nat. Biotechnol.* 32(8):760–772, 2014. 25093883 10.1038/nbt.2989

[118] Moya-Rodriguez, A., et al. Creating patient-specific vein models to characterize wall shear stress in hemodialysis population. *Comput. Struct. Biotechnol. J.* 20:5729–5739, 2022. 36382195 10.1016/j.csbj.2022.10.010 PMC9619312

[119] Huynh, T. N., et al. Effects of venous needle turbulence during ex vivo hemodialysis on endothelial morphology and nitric oxide formation. *J. Biomech.* 40(10):2158–2166, 2007. 17161843 10.1016/j.jbiomech.2006.10.028

[120] Shah, N. A., et al. PRINTcision medicine: a reusable, three-dimensional-printed, patient-specific, in vitro model of arteriovenous fistulas for endothelial cell studies. *J. Am. Soc. Nephrol.* 2024. 10.1681/ASN.2024t9n1z2ex.38640019

[121] Kwei, S., et al. Early adaptive responses of the vascular wall during venous arterialization in mice. *Am. J. Pathol.* 164(1):81–89, 2004. 14695322 10.1016/S0002-9440(10)63099-4 PMC1602233

[122] Wong, C. Y., et al. Vascular remodeling and intimal hyperplasia in a novel murine model of arteriovenous fistula failure. *J. Vasc. Surg.* 59(1):192–201 e1, 2014. 23684425 10.1016/j.jvs.2013.02.242

[123] Wong, C. Y., et al. Elastin is a key regulator of outward remodeling in arteriovenous fistulas. *Eur. J. Vasc. Endovasc. Surg.* 49(4):480–486, 2015. 25701072 10.1016/j.ejvs.2014.12.035

[124] Bezhaeva, T., et al. Relaxin receptor deficiency promotes vascular inflammation and impairs outward remodeling in arteriovenous fistulas. FASEB J. 2018. 10.1096/fj.201800437r. 29882709 10.1096/fj.201800437R

[125] Yang, B., et al. The mouse arteriovenous fistula model. *J. Vasc. Interv. Radiol.* 20(7):946–950, 2009. 19555889 10.1016/j.jvir.2009.03.044

[126] Yamamoto, K., et al. Technical aspects of the mouse aortocaval fistula. *J. Vis. Exp.* 77:e50449, 2013. 10.3791/50449 PMC3732098 23892387

[127] Yamamoto, K., et al. The mouse aortocaval fistula recapitulates human arteriovenous fistula maturation. *Am. J. Physiol. Heart Circ. Physiol.* 305(12):H1718–H1725, 2013. 24097429 10.1152/ajpheart.00590.2013 PMC3882542

[128] Karram, T., et al. Induction of cardiac hypertrophy by a controlled reproducible sutureless aortocaval shunt in the mouse. *J. Investig. Surg.* 18(6):325–334, 2005. 16319054 10.1080/08941930500328839

[129] Kudze, T., et al. Altered hemodynamics during arteriovenous fistula remodeling leads to reduced fistula patency in female mice. *JVS Vasc. Sci.* 1:42–56, 2020. 32754721 10.1016/j.jvssci.2020.03.001 PMC7402599

[130] Cui, J., et al. Atorvastatin reduces in vivo fibrin deposition and macrophage accumulation, and improves primary patency duration and maturation of murine arteriovenous fistula. *J. Am. Soc. Nephrol.* 31(5):931–945, 2020. 32152232 10.1681/ASN.2019060612 PMC7217409

[131] Chan, C. Y., et al. Remodeling of experimental arteriovenous fistula with increased matrix metalloproteinase expression in rats. *J. Vasc. Surg.* 45(4):804–811, 2007. 17398390 10.1016/j.jvs.2006.12.063

[132] Bai, H., et al. Artery to vein configuration of arteriovenous fistula improves hemodynamics to increase maturation and patency. *Sci. Transl. Med.* 12(557):eaax7613, 2020. 32817365 10.1126/scitranslmed.aax7613 PMC7705473

[133] McEnaney, R. M., D. McCreary, and E. Tzeng. A modified rat model of hindlimb ischemia for augmentation and functional measurement of arteriogenesis. *J. Biol. Methods* 5(2):e89, 2018. 31435496 10.14440/jbm.2018.234 PMC6703558

[134] Andraska, E., et al. Simultaneous upregulation of elastolytic and elastogenic factors are necessary for regulated collateral diameter expansion. *Front. Cardiovasc. Med.* 8:762094, 2021. 35096993 10.3389/fcvm.2021.762094 PMC8789883

[135] Yang, L., et al. Endothelialization of PTFE-covered stents for aneurysms and arteriovenous fistulas created in canine carotid arteries. *Sci. Rep.* 14(1):4803, 2024. 38413764 10.1038/s41598-024-55532-5 PMC10899654

[136] Rogers, K. M., M. J. Merrilees, and W. E. Stehbens. The effect of haemodynamic stress on the glycosaminoglycan content of blood vessel walls of experimental aneurysms and arteriovenous fistulae. *Atherosclerosis* 58(1–3):139–148, 1985. 4091877 10.1016/0021-9150(85)90061-9

[137] Boudghene, F., et al. Aortocaval fistulae: a percutaneous model and treatment with stent grafts in sheep. *Circulation* 94(1):108–112, 1996. 8964110 10.1161/01.cir.94.1.108

[138] Rotmans, J. I., et al. Rapid, arteriovenous graft failure due to intimal hyperplasia: a porcine, bilateral, carotid arteriovenous graft model. *J. Surg. Res.* 113(1):161–171, 2003. 12943826 10.1016/s0022-4804(03)00228-2

[139] Rodriguez, V. M., et al. Effects of brachytherapy on intimal hyperplasia in arteriovenous fistulas in a porcine model. *J. Vasc. Interv. Radiol.* 13(12):1239–1246, 2002. 12471188 10.1016/s1051-0443(07)61971-x

[140] Barreiro, K. A., et al. Novel locally acting dual antiplatelet and anticoagulant (APAC) targets multiple sites of vascular injury in an experimental porcine model. *Eur. J. Vasc. Endovasc. Surg.* 58(6):903–911, 2019. 31708337 10.1016/j.ejvs.2019.05.019

